# The Retornus-2 study: impact of respiratory muscle training in subacute stroke patients with dysphagia, study protocol of a double-blind randomized controlled trial

**DOI:** 10.1186/s13063-021-05353-y

**Published:** 2021-06-25

**Authors:** A. Guillen-Sola, M. Messaggi-Sartor, C. Ramírez-Fuentes, E. Marco, E. Duarte

**Affiliations:** 1 Department of Physical Medicine and Rehabilitation, Parc de Salut Mar (Hospital del Mar-Hospital de l’Esperança), Barcelona, Spain; 2 Rehabilitation Research Group, Hospital del Mar Research Institute, Barcelona, Spain

**Keywords:** Stroke, Randomized clinical trial, Dysphagia, Respiratory muscle training, Rehabilitation

## Abstract

**Background:**

Stroke can lead to varying degrees of oropharyngeal dysphagia, respiratory muscle dysfunction and even increase medical complications such as aspiration, malnutrition and death. Recent studies suggest that inspiratory and expiratory respiratory muscle training (IEMT) can improve swallowing efficacy and may reduce aspiration events. The main purpose of this study is to examine whether an 8-week IEMT programme can improve respiratory muscle strength and swallow dysfunction severity in subacute stroke patients with dysphagia.

**Methods:**

Retornus-2 is a two-arm, prospectively registered, randomized controlled study with blinded assessors and the participation of fifty individuals who have suffered a stroke. The intervention group undergoes IEMT training consisting of 5 sets of 10 repetitions, three times a day for 8 weeks. Training loads increase weekly. The control group undergoes a sham-IEMT protocol. The primary outcome examines the efficacy of the IEMT protocol to increase respiratory muscle strength and reduce dysphagia severity. The secondary outcome assesses the longitudinal impact of dysphagia on body composition and nutritional assessment over a 6-month follow-up.

**Discussion:**

IEMT induces an improvement in respiratory muscle strength and might be associated with relevant benefits in dysphagia patterns, as well as a reduction in the number of aspiration events confirmed by videofluoroscopy or fiberoptic endoscopic evaluation of swallowing. The description of the impact of swallowing impairment on nutritional status will help develop new strategies to face this known side-effect.

**Trial registration:**

Clinicaltrials.gov NCT03021252. Registered on 10 January 2017. https://clinicaltrials.gov/ct2/results?cond=retornus+2&term=&cntry=ES&state=&city=&dist=

WHO trial Registration data set: Due to heavy traffic generated by the COVID-19 outbreak, the ICTRP Search Portal does not respond. The portal recommends other registries such as clinicaltrials.gov. Protocol version: RETORNUS 2_ PROTOCOL_2.

**Supplementary Information:**

The online version contains supplementary material available at 10.1186/s13063-021-05353-y.

## Introduction

Stroke is one of the major causes of disability in adults, being an important public health concern [1]. Stroke-associated pneumonia is a well-documented complication occurring after the first months of the event. The incidence of aspiration pneumonia in medical wards and rehabilitation units ranges from 3.9 to 45%, with a median rate of 7.4% [2].

Breathing, eating and cough function play an important role in the prevention of aspirations and consequent respiratory complications. Regulator mechanisms are located on the brainstem [3, 4] and coordinate the motor activity and appropriate response to sensory feedback.

The prevalence of respiratory muscle weakness in subacute stroke is very high [5]. After a stroke, the muscular dysfunction and the lack of coordination secondary to central nervous system damage may lead to the development of dysphagia [6]. The incidence of dysphagia is high among the stroke population, ranging between 19 and 81%; these differences in dysphagia incidence could be attributed to variations in the method of identification, assessment time from stroke onset and lesion location [7]. Considering the variability of outcomes to stroke-related dysphagia, to improve protocols for an early detection and to provide an efficient treatment might reduce respiratory complications and reduce other health care expenditures [8].

Over the last decade, a novel non-pharmacological approach to respiratory complications has been developed and tested in several neurological diseases. Positive results have been observed using inspiratory and expiratory muscle training (IEMT) to improve cough effectiveness and airway protection in neurological patients [9–14]. To date, several randomized clinical trials in different neurological conditions have demonstrated significant improvements in respiratory muscle function and other physiologic parameters after IEMT [14–17].

Focusing on stroke patients, some authors tested a dual training programme for inspiratory and expiratory muscle training (IEMT) and concluded that IEMT induces significant improvement in inspiratory and expiratory muscle strength. This could potentially offer an additional therapeutic tool aimed at reducing respiratory complications at 6 months in stroke patients [5] while providing class II evidence of this practice. Other authors reported positive results on dyspnea parameters in people with post-stroke respiratory muscle weakness [14]. A recent systematic review conducted by Menezes et al. [10] concluded that 30 min of IEMT, five times a week, for 5 weeks increases respiratory muscle strength in very weak individuals after stroke, reducing the risk of respiratory complications. Nevertheless, we can also find conclusions with the opposite view, reporting that respiratory function and cough parameters improve within 2 weeks of stroke onset and IEMT does not expedite this improvement [17].

IEMT is also a therapeutic strategy to be considered in patients with dysphagia mainly because of the effect of expiratory muscle training (EMT) on suprahyoid muscles and constrictors. EMT reduces the pharyngeal residue in Parkinson disease [18] by improving pharyngeal hypokinesia and swallowing efficiency. IEMT also improves pharyngeal swallowing security signs after stroke [16].

Under the hypothesis that IEMT could improve respiratory and swallow function in subacute stroke, a randomized clinical trial is designed to assess the effectiveness of an 8-week IEMT programme to improve respiratory muscle strength and swallowing function in subacute stroke patients with dysphagia.

The Retornus-2 study has been designed as a parallel-group double-blind randomized controlled trial with allocation ratio 1:1 in a superiority framework of the intervention and intention-to-treat analyses. The study will follow the Standard Protocol Items: Recommendations for Interventional Trials (SPIRIT) guidelines [19].

## Methods

### Study setting

The study is carried out in the Neurorehabilitation Unit, Physical Medicine and Rehabilitation Department, of a tertiary hospital in Barcelona (Parc de Salut Mar, Barcelona, Catalonia, Spain).

### Eligibility criteria

The population of interest in this study is comprised of stroke outpatients with oropharyngeal dysphagia. The inclusion criteria for the study are (1) oropharyngeal dysphagia diagnosed by a videofluoroscopic swallow study (VFSS) or fiberoptic endoscopic swallow study (FEES) secondary to first ischemic stroke, (2) stroke onset >3 weeks and (3) preserved cognitive function (MMSE >24). Exclusion criteria include (1) motor or sensitive aphasia which could impair complete understanding of exploratory items; (2) serious cardiovascular events (non-clinically stable condition, recent heart failure or recent changes in cardiac function), neuromuscular diseases (neurodegenerative diseases) or metabolic myopathies conditions that could interfere with the results and/or measurements; and (3) medicines with potential effect on muscle structure and function (steroids, thyroid hormones, immunosuppressants).

### Interventions

The intervention consists of an IEMT protocol using the Orygen Dual Valve (Forumed S.L, Barcelona, Catalonia, Spain). This device allows patients to train inspiratory and expiratory muscles simultaneously, and loads can be adjusted independently at regular intervals of 10 cmH_2_O. Training load was set to the inspiratory and expiratory pressures allowing patients to perform 10 maximal repetitions (RM), and adjusted weekly by 10 cmH_2_O if tolerated. Patients performed five sets of 10 inspirations and expirations, three times a day, 7 days per week, for 8 weeks. The control group received the same training schedule using a sham valve. Patients of both groups received standard therapy consisting of swallowing manoeuvres, oral exercises and compensatory techniques aimed at improving self-management of dysphagia and protect the airway. The training session was supervised by a Speech and Language Therapist (SLT) twice a week, and the other sessions were self-assisted at home.

There are no special criteria for discontinuing or modifying allocated interventions. Patients requiring discontinuing or modifying the interventions were withdrawn from the study. Strategies to improve adherence to respiratory training included providing a record sheet to register the home training sessions. Concomitant SLP therapy or respiratory therapies (oxygen therapy, breathing treatments, humidity-aerosol therapy, pulmonary drainage procedures, respiratory exercises, exercises with a triflow device…) were not permitted during the trial.

### Study outcomes

Two primary *outcomes* are contemplated: strength of the inspiratory and expiratory muscles after an 8-week IEMT protocol, estimated with maximal inspiratory and expiratory pressures (PImax and PEmax, respectively) expressed in cmH_2_O, and dysphagia severity assessed by VFSS. Secondary outcomes are voluntary peak cough flow (measured using a PEFR meter) and tongue strength (measured with the IOPI® system). All data was collected at baseline, end-treatment and 6-month follow-up.

Respiratory complications were also registered during the follow-up by asking the participants and were checked in the personal health history if whether they had been admitted to a hospital (Public Health System, on-line access) for respiratory causes (e.g. pneumonia or lung infections).

### Participant timeline

**Fig. 1:**
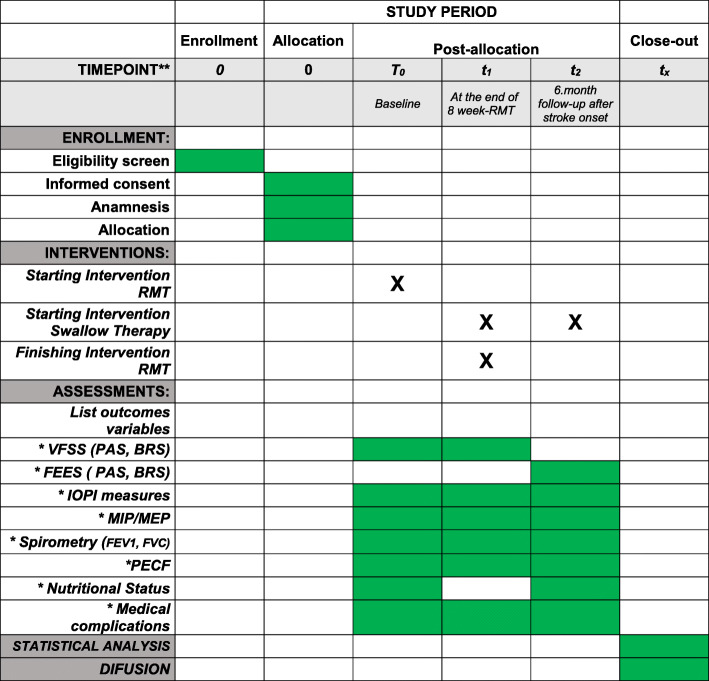
Standard Protocol Items: Recommendations for Interventional Trials (SPIRIT). Schedule of enrollment, interventions and assessments

The principal investigator screened the eligibility of potential participants during hospital admission as of April 2017. These patients received detailed information about the study procedures and were invited to participate. Demographic and clinical characteristics of the participants were collected at baseline (T0). Primary and secondary outcomes were assessed at baseline (T0), after 8 week-exercise intervention (T1) and 6-month follow-up (T2), as shown in Fig. 1.

### Sample size

Accepting an alpha risk of 0.05 and a beta risk of 0.2 in a two-sided test, 25 patients are required in each group to detect a difference of 27 (SD 31) cmH_2_O in PImax, anticipating potential losses of 15% (final size 50 patients). These assumptions have been based on data of de Menezes et al. [14]. The sample size was calculated using the GRANMO Sample Size and Power Calculation software of the Hospital del Mar Research Institute (IMIM).

### Recruitment

Patients admitted in the Neurorehabilitation unit received detailed information about the study procedures. All members of staff and health professionals involved with the care of patients were also informed and asked to encourage patients to participate. Informed consent was obtained just before discharge by a team researcher.

### Allocation

This trial was designed as a parallel-group double-blind randomized controlled trial with allocation ratio 1:1 in a superiority framework of the intervention and intention-to-treat analyses. Allocation to study groups was performed independently by a member of the medical staff blinded to patient identity using a randomization programme designed by the IT Department. Patients and researchers in charge of assessments were blinded to study group assignments. Patients were randomly allocated to either an intervention (IEMT) group or a control (sham) group. Allocation concealment was ensured as the person in charge of randomization did not release the randomization codes until participants completed all measurements. Training began at hospital discharge. Outcome measures were collected by a trained research worker at baseline (week 0), at the end of training (week 8) and 6 months after cessation of training. Data collection and analysis are carried out by a research worker blinded for the allocation group.

**Fig. 2:**
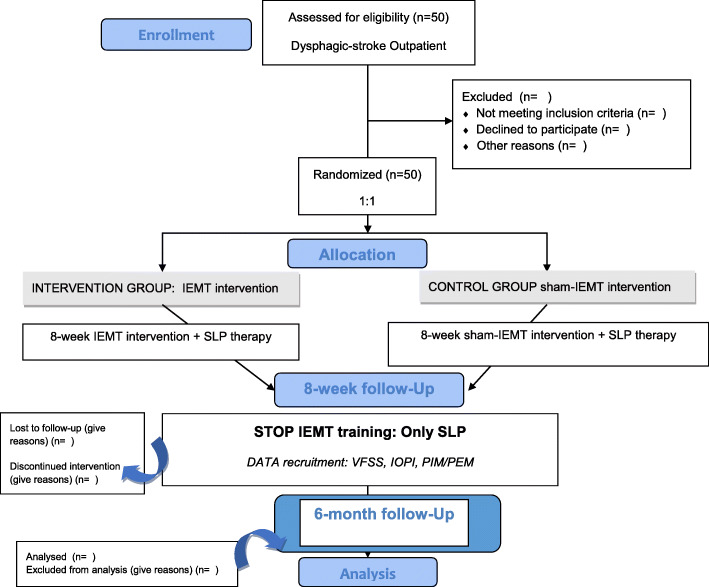
Flow chart

Study sites and phases are summarized in the flow chart diagram (Fig. 2).

### Blinding

Participants and therapists were blinded for the assignments, as well as researchers in charge of data assessment recruitment. Requirements for unblinding were not anticipated, but if required, the trial manager, data coordinator and clinicians had access to group allocations and any unblinding were reported.

Once data analysis will be completed, the results will be released to all patients and to participating clinicians and researchers.

### Data collection, management and analysis

*Instruments for swallowing and respiratory function study:*

1. *Swallowing function parameters:*
  1.1. *Videofluoroscopy* was selected for examination of swallowing function. The lateral profile sequence of fluoroscopy was captured by continuous recording with digital storage of images at a rate of 25 frames per second. Visipaque®, a water-soluble contrast, and Thickener Resource®, a ThickenUp, were used to obtain 3 distinct viscosities (liquid, nectar, pudding). Water-soluble contrast is available in bottles of 50 mL (16 mg iodine) or 320 mg/mL. Liquid viscosity was obtained by mixing 1:1 mineral water and water-soluble contrast at room temperature (100 mL/16mg iodine); thickener was added to the liquid solution in a mixing pan to obtain nectar and pudding viscosity (3.5 g and 8 g of thickener, respectively). Solutions were prepared 10 min before measurement. For each viscosity and soft solid, boluses in increasing volumes of 5, 10 and 20 mL were offered to patients with a syringe. The worst punctuation obtained regardless of the altered viscosity was determined on the Penetration-Aspiration Scale [20] and the amount of residue generated on the Bolus Residue Scale (BRS) [21]. Both values were recorded for statistical analysis.
  1.2. *Fiberoptic endoscopic evaluation of swallowing, FEES*: At 6 months after the stroke event, swallowing function was assessed using a standardized FEES examination. During FEES, patients are seated in an upright position and a flexible endoscope (Xion system©) introduced into the inferior turbinate in the inferior meatus; it will then be passed through the oropharynx to a point posterior to the epiglottis where the general appearance of the laryngeal structures can be clearly visualized. The patient is asked to phonate, to enable assessment of the adequacy of vocal fold adduction. At this point, three consistencies were administered for consecutive trials of 5/10/20 cc of thick liquid (applesauce dyed with green food colouring), 5/10/20 cc of thin liquid (water dyed with 5% methylene blue) and 5 ml of pudding bolus. The bolus consistencies were administered in the same sequence for all patients. FEES images were obtained and recorded on a DVD [22]. A Penetration-Aspiration Scale (PAS) [23, 24] was used to detect entry or residue. The worst punctuation obtained on each scale, regardless of the altered viscosity, was recorded for statistical analysis.
  Both techniques were performed by a 10-year experienced senior medical staff, blinded for the training programme allocation.
  1.3. *The IOPI® system was used to measure tongue function.* Tongue strength and peak pressure were obtained by measuring the amount of pressure exerted by the tongue on an air-filled bulb attached to a pressure transducer. A peak holding circuit displays peak pressure on a digital readout in kilopascals (kPa). Calibration was checked once a week as recommended in the IOPI manual to ensure the accuracy of the measurement. As no population data was available from Spain or Catalonia, Belgian population data will be used to check the results [25].
2. *Respiratory function parameters:*
  2.1. *Respiratory muscle pressures were determined by asking* patients to perform maximum inspiratory pressure (MIP) from residual volume and maximum expiratory pressure (MEP) from total lung capacity; both manoeuvres were performed against an occluded airway. The mouthpiece used in the manoeuvres has a small orifice to minimize the participation of face and mouth muscles and is connected to a pressure transducer Micro RPM (Micro Medical/CareFusion, Kent, UK). A flanged mouthpiece is used to create an optimal mouth seal. The highest value of 3 reproducible manoeuvres (10% variability between values) is used for analysis and expressed as a percentage of the reference values determined for a Mediterranean Caucasian population [26]. Based on published research assessing respiratory muscle strength, inspiratory and expiratory muscle weakness was defined as PImax and PEmax, being 70%, respectively, of predicted values [27, 28].
  2.2. *Respiratory function tests:* Respiratory testing was performed with the participant seated comfortably. Spirometry -(forced expiratory volume at 1 second (FEV1), forced vital capacity (FVC) and peak expiratory flow rate (PEF) - were assessed using a portable spirometer (SpiroUSB; CareFusion, San Diego, CA, USA) and a bacterial filter (Spiroguard Standard; Air Safety Medical, Morecambe, England). A flanged mouthpiece (Rubber Flanged Mouthpiece MTH6400; CareFusion) was used to create an optimal mouth seal in the presence of orofacial weakness.
  2.3. *Peak expiratory cough flow:* Each participant was asked to complete five-strong coughs into an analogue PEFR meter (Mini-Wright Peak Flow Meter). The highest value of the 5 reproducible manoeuvres (10% variability between values) was considered for analysis.
  A team researcher was responsible for data recruitment, blinded for the training programme allocation. SLP was responsible for training programme, blinded for allocation.

### Data collection methods

The main outcomes were collected by means of a Data Collection Logbook specially designed for this study. This logbook included the outcome measures previously described. One member of the investigation team was responsible for the collection and processing of data related to main outcome variables. To promote participant retention, patients performed 2 weekly supervised sessions; any patient not attending these scheduled sessions was contacted by telephone.

### Data management

Two researchers were in charge of the database. On the basis of the information collected on the Data Collection sheets, one of them entered data into the database, and the second researcher checked data accuracy. No personally identifying information was registered and a code number was assigned to each participant. A list with code numbers was used to randomize patients to control or intervention groups by an external researcher not involved with the trial procedures.

### Statistical analysis

Descriptive statistics summarizes the demographic and clinical outcomes of the participants. Assumption of normality is analysed using normality charts and the Kolmogorov-Smirnov test corrected with the Lilliefors test. The bivariate analysis is performed using the chi-square, Fisher exact, Student t or Mann-Whitney U tests, depending on the variables analysed.

Treatment effect is analysed in two ways: changes in PImax and PEmax pre- and post-intervention (ΔPImax and ΔPEmax, respectively) and by calculating the percentage change using the formula [((post value − pre-value) /pre-value) × 100]. Changes during follow-up are assessed by analysis of variance using mixed repeated measures and a one-factor design for the analysis of values over time.

All statistical tests are two-tailed and statistical significance has been set at 0.05. IBM SPSS 22.0 software is used for statistical analysis. The final report will follow CONSORT 2010 guidelines.

When estimating the study’s sample size, the possibility of loss to follow-up was considered and factored into the calculation. Moreover, other missing data was accounted for by handling drop-outs as non-finishing intervention, in accordance with the intention-to-treat principle.

### Data monitoring and harms

Given the non-pharmacological nature of this study, a data monitoring committee is not required. Any adverse effect related to the exercise intervention or any unintended effect was registered. There are no anticipated problems that could be detrimental to the participant. Interim analyses are not contemplated, but if required, only the main investigators will be involved.

### Steering committee and audits

No steering committee was constituted. The principal investigators supervised correct development of the trial. A monthly meeting was held to detect any eventual difficulties or errors. Both investigators were responsible for the design of the trial but did not participate in data recruitment or intervention. They met with the intervention group every 6 months, to detect and correct any eventual difficulties during the recruitment and follow-up processes, data management and monitoring and statistical analysis of outcomes. They were responsible for assessing the rate of progress to ensure that the trial was conducted in accordance with the study plan.

No intermediate audits were contemplated. But should any unexpected adverse effects appear, audit process will be carried out in order to detect and correct the side-effects.

## Ethics and dissemination

### Research ethics approval and consent

National and international research ethics guidelines were followed, including the Deontological Code of Ethics, Declaration of Helsinki and current confidentiality laws concerning personal data in Spain (Organic Law 3/2018, December 5th) and the European Union *(European Parliament and Council Regulation EU 2016/619)*. Detailed, understandable oral and written information was provided to patients and family members, and an informed consent to participate will be signed by all participants (Additional File 1 A and B). The Retornus-2 study protocol and the informed consent process were reviewed and approved by the Clinical Ethics Committee of the *Institut Hospital del Mar d´Investigacions Mèdiques*, Barcelona, Spain (*Comité Ètic d’Investigació Clínica Parc de Salut Mar*: reference number 2016/6796/I) (Additional file 2).

Prior to any intervention, each patient was informed about the risk and the goals of the current study. Patients were allowed to withdraw from the study at any time for any reason. Patients received the required treatment regardless of participation in the study.

### Protocol amendments

Participants, the research team and trial registries were notified of any eventual modifications of the study protocol.

*Confidentiality* included in data management

*Declaration of interest/Competing interests* (included in the Declarations section)

*Access to data/Availability of data and materials* (included in the Declarations section)

*Ancillary and post/trial care* (included in the Declarations section)

### Dissemination policy

The findings of this research project will be disseminated nationally and internationally in scientific meetings, and we will seek publication in peer-reviewed journals.

## Discussion

This study is an attempt to evaluate the effect of IEMT on respiratory muscle function and dysphagia severity. Adherence to the 8-week protocol was ensured by using an external device for training. Retornus-2 is the natural follow-up of the first study of our group, the Retornus I study, in which we reported that inspiratory and expiratory muscle training (IEMT) induces significant improvement in inspiratory and expiratory muscle strength and could potentially offer an additional therapeutic tool aimed at reducing respiratory complications at 6 months in stroke patients [5]. Secondly, adding IEMT to Standard Swallowing Therapy (SST) was effective and feasible and provided a safe approach to improving respiratory muscle strength and swallow function [16]. This second study is the result of the difficulties encountered in implementing the training protocol in acute patients (Retornus-1). The main difference between trials was to train the patient during admission in rehabilitation (acute post-stroke phase, Retornus-1) or at discharge when the patient returned home (> 1 month post-stroke event, Retornus-2). The authors consider that this trial will contribute evidence for the development of new strategies for accelerating recovery from dysphagia and the use of external devices.

In conclusion, the results of the trial may result in important advances in neurological dysphagia rehabilitation. First, the magnitude of effect on dysphagia may be higher than previous trials, where the focus is placed on improving respiratory strength. Second, the trial tests the capacity of patients for training at home, facilitating rehabilitation intervention. Finally, if the results lead to a reduction in hospital admissions, respiratory muscle training could help reduce direct and indirect costs associated with stroke.

### Strengths and limitations of the study

Samples from rehabilitation units usually have an initial bias because patients tend to be preselected for their potential to follow a rehabilitation programme. This is an initial bias due to the fact that not all the stroke patients are admitted to follow an intensive rehabilitation programme and some of them are transferred to other facilities (e.g. nursing home). Patients in our study were recruited during their stay in the neurorehabilitation ward (intensive rehabilitation). Once functional status was enough to return home, our Health System provided transport facilities to continue the rehabilitation programme.

The decision to only evaluate patients in a subacute stroke phase may provide limited results and make extrapolation to other populations difficult. The homogeneity of participants due to selected-dysphagic post-stroke events impacts on the applicability of our findings to the general stroke population but not for a population similar to the sample described in this study.

## Trial status

*Protocol version number:* Retornus-2_2

*Begin recruitment:* 05/07/2017

*End recruitment:* 03/31/2020

*End data collection:* 09/31/2020

*Trial status:* Enrolment is completed

*Statement about trial status:* Because of the COVID-19 pandemic, enrolment was closed in March 2020 with 48 patients (in sample size calculation, an overhead of 15% was calculated). The follow-up ended in September 2020.

## Supplementary Information

**Additional file 1.** A: Informed consent. B: Informed consent.

**Additional file 2.** Ethical Approval Document

**Additional file 3.** SPIRIT checklist.

**Additional file 4.** Funding Documentation.

## Data Availability

The datasets generated and/or analysed during the current study are not publicly available due to the amount of data generated but are available from the corresponding author on reasonable request.

## Abbreviations

RMT: Respiratory muscle training
IEMT: Inspiratory and expiratory muscle training
EMT: Expiratory muscle training
VFSS: Videofluoroscopy swallowing study
RCT: Randomized control trial
MIP: Maximum inspiratory pressure
MEP: Maximum expiratory pressure
SSP: Swallowing and speech pathologist
