# Supplementary material for: The Retornus-2 study: impact of respiratory muscle training in subacute stroke patients with dysphagia, study protocol of a double-blind randomized controlled trial
Source: Trials. 2021 Jun 25;22:416. doi: 10.1186/s13063-021-05353-y (PMC8229262; doi:10.1186/s13063-021-05353-y)
Supplement: Supplementary file 1 — Additional file 1. A: Informed consent. B: Informed consent. [file 13063_2021_5353_MOESM1_ESM.zip › 4 b_ADDITIONAL FILE 1A_CONSENTIMENT INFORMAT_CATR2.pdf]

FULL D'INFORMACIÓ i CONSENTIMENT INFORMAT

"Estudi RETORNUS-2: Impacte de l'Entrenament respiratori en la funció deglutòria en pacientes amb disfàgia secundària a ictus"

Els metges del Servei de Rehabilitació del Parc de Salut Mar (Hospital del Mar- Hospital de l'Esperança) volen conèixer quins paràmetres determinen la resposta a diferents programes d'entrenament en pacients, que com vostè, poden presentar debilitat de la musculatura respiratòria i deglutòria.

Vostè ha estat seleccionat per participar en aquest estudi per presentar dificultat en la capacitat de deglutir/empassar els aliments com a conseqüència d'un ictus. L'objectiu de l'estudi és avaluar el benefici que pot aportar l'entrenament de la musculatura respiratòria en l'evolució de la malaltia.

Amb aquest full informatiu , volem sol·licitar la seva participació en aquest estudi, oferint tota la informació necessària perquè pugui prendre lliurement la decisió d'acceptar o rebutjar la participació voluntària en aquest estudi. En aquest sentit cal informar-lo que la informació i la sol·licitud del consentiment informat són normatius i d'obligat compliment per l'equip mèdic que l'atendrà. Si accepta a participar en aquest estudi suposarà un doble benefici, tant per vostè com per altres pacients. L'estudi ens permetrà conèixer el tractament més adequat per cada pacient.
L'objectiu del document és fer-lo coneixedor dels detalls de les proves diagnòstiques que se li faran abans d'iniciar l'estudi. Aquest procés requereix dels eu consentiment informat.

RESUM DE L'ESTUDI

L'entrenament muscular sembla millorar la funció de la musculatura deglutòria i respiratòria, però encara resta per definir els pacients que es poden beneficiar d'una pauta específica d'entrenament.
El títol de l'estudio és *"Estudio RETORNUS-2: Impacte de l'Entrenament respiratori en la funció deglutòria en pacients amb disfàgia secundària a ictus"*
A part de les proves convencionals que es realitzaran pel seu diagnòstic i posterior tractament , es realitzarà una avaluació específica de la força de la musculatura deglutòria i respiratòria.
L'entrenament que vostè realitzarà consisteix en exercicis de respiració a través d'una vàlvula calibrada segons el seu nivell de tolerància i durarà 8 setmanes. L'entrenament es realitzarà en sessions setmanals d'aproximadament 45 minuts i posteriorment a domicili.

AL·LEATORITZACIÓ

Tots els pacients rebran entrenament respiratori però pot variar la intensitat . LA duració serà de 8 setmanes i l'assignació a cada grup es realitzarà per sorteig .

PRIVACITAT

Durant l'estudi se seguiran les directrius nacionals i internacionals (Codi Deontològic, Declaració de Helsinki) per la investigació amb éssers humans. Tot el personal que participa en l'estudio respectarà la seva intimitat. La seva història clínica és confidencial. El seu nom i dades personals no seran proporcionats a persones no autoritzades (Llei Orgànica 15/1999 de 13 de desembre) de Protecció de Dades de Caràcter Personal (LOPD).

DRET a INTERROMPRE L'ESTUDI: La decisió de participar en l'estudio és del pacient. Els metges que participiran tenen el deure de salvaguardar en tot moment el benestar del pacient i vigiliran perquè tingui les mínimes molèsties. Si té cap dubte, ha de preguntar-lo al metge responsable. Si decideix interrompre l'estudi en algun moment, es troba en plena llibertat de fer-ho sempre i quan desitgi.

Els professionals responsables de l'estudi són:

- Servei de Medicina Física i Rehabilitació: Dra. Anna Guillén-Solà Dra. Esther Duarte, Sra. Monique Messaggi-Sartor

- Qualsevol d'ells està capacitat per respondre a les preguntres que vostè precisi aclarir .

Jo (nom i cognoms) ____ declaro que:
- He llegit el full d'informació de l'estudi que se m'ha entregat
- He pogut realitzar preguntes sobre l'estudi
- He rebut informació suficient sobre l'estudi
- He estat informat per (nom de l'investigador) _________________________

Comprenc que la meva participació és voluntària i que puc retirar-me de l'estudi:
- Quan vulgui
- Sense donar explicacions
- Sense repercussions en la meva atenció mèdica

Declaro haber estat informat que les meves dades seran tractades conforme el que estableix la llei L.O. de 13 de desembre i de Protecció de Dades de Caràcter Personal (artícle 3, punt 6 del RD 223/2004).
Consenteixo que les dades clíniques referents a la meva malaltia siguin emmagatzemats en un fitxer informàtic per poder ser explotat amb finalitats exclusivament científiques.

Per aquest motiu: dono el meu consentiment a participar en l'estudi que se m'ha proposat

Firma del metge responsable — Firma del voluntari

Dr/a.: — Sr/a.:
Núm. Col.:
Data :

He decidit no autoritzar a participar en l'estudi que se m'ha proposat

Firma del metge responsable — Firma del voluntari

Dr/a.: — Sr/a.:
Núm. Col.:
Data:

CONSORCI MAR PARC DE SALUT DE BARCELONA I NIF: S-0800471E PAPER ECOLÒGIC

Sant Josep de la Muntanya,12 I 08024 Barcelona I Telèfon 933 674 100 I Fax 933 674 266
www.parcdesalutmar.cat
