# Supplementary material for: The Retornus-2 study: impact of respiratory muscle training in subacute stroke patients with dysphagia, study protocol of a double-blind randomized controlled trial
Source: Trials. 2021 Jun 25;22:416. doi: 10.1186/s13063-021-05353-y (PMC8229262; doi:10.1186/s13063-021-05353-y)
Supplement: Supplementary file 1 — Additional file 1. A: Informed consent. B: Informed consent. [file 13063_2021_5353_MOESM1_ESM.zip › 4_ADDITIONAL FILE 1B_CONSENTIMENT INFORMAT_CASTR2.pdf]

HOJA DE INFORMACIÓN Y CONSENTIMIENTO INFORMADO

"Estudio RETORNUS-2: Impacto del Entrenamiento respiratorio en la función deglutoria en pacientes con disfagia secundaria a ictus"

Los médicos del Servicio de Rehabilitación del Parc de Salut Mar (Hospital del Mar y Hospital de l'Esperança) se encuentran interesados en conocer qué parámetros determinan la respuesta a diferentes programas de entrenamiento en pacientes que, como Usted, pueden presentar debilidad de los músculos deglutorios y respiratorios.

Usted ha sido seleccionado para participar en este estudio por presentar algún grado de dificultad en la delgución de alimentos como consecuencia del ictus. El objetivo del estudio es evaluar el beneficio que puede aportarle a usted el entrenamiento de músculos respiratorios en la evolución de su enfermedad.

El propósito de esta información es solicitarle la participación en este estudio, ofreciendo la información necesaria para que usted pueda tomar la decisión, libre y voluntaria, de autorizar o rechazar la participación como voluntario en este estudio. En este sentido le comunicamos que informarle y solicitar su autorización es una norma de obligado cumplimiento para el/la médico que le atiende.
Si acepta a participar en este estudio supondrá un doble beneficio, tanto para Usted como para otros enfermos. Este estudio nos permitirá conocer mejor cual es el tipo de tratamiento más adecuado para cada paciente.
El objetivo de este documento es que Usted conozca los detalles de las maniobras diagnósticas que se le practicarán, antes de decidir su participación en este estudio. Este proceso se denomina consentimiento informado.

RESUMEN DEL ESTUDIO
El entrenamiento muscular parece mejorar la función de los músculos deglutorios y respiratorios, sin embargo no está bien definido qué pacientes se pueden beneficiar de una pauta específica de entrenamiento.
El título del estudio es *"Estudio RETORNUS-2: Impacto del Entrenamiento respiratorio en la función deglutoria en pacientes con disfagia secundaria a ictus"*
Además de las pruebas convencionales que se realizan para su diagnóstico y posterior tratamiento, se realizará una evaluación específica de la fuerza de los músculos deglutorios y respiratorios.
El entrenamiento que usted realizará consiste en ejercicios de respiración a través de una válvula en la cual colocaremos pesos progresivamente según su nivel de tolerancia, durante 8 semanas. Todo ello se realizará en sesiones semanal de aproximadamente 45 minutos y posterior trabajo en domicilio.

ALEATORIZACIÓN
Todos los pacientes recibirán entrenamiento respiratorio, pero puede variar su intensidad. La duración será de 8 semanas y la asignación a un grupo u otro de tratamiento se realizará por sorteo.

PRIVACIDAD
Durante el estudio se seguirán las directrices nacionales e internacionales (Código Deontológico, Declaración de Helsinki) para la investigación en seres humanos. Todo el personal que participa en el estudio respetará su intimidad. Su historia clínica es confidencial. Su nombre y demás datos personales no serán proporcionados a personas no autorizadas (Ley Orgánica 15/1999 de 13 de diciembre) de Protección de Datos de Carácter Personal (LOPD).

DERECHO DE INTERRUMPIR EL ESTUDIO: La decisión de participar en el estudio es del paciente. Los médicos que participarán en el mismo salvaguardarán en todo momento su bienestar y velarán porque tenga el mínimo de molestias. Si tiene alguna duda debe preguntar a su médico. Si decide interrumpir el estudio en algún momento, se halla en plana libertad de hacerlo siempre y cuando lo desee.

Los profesionales responsables del estudio son:

- Servicio de Medicina Física y Rehabilitació: Dra. Anna Guillén-Solà Dra. Esther Duarte, Sra. Monique Messaggi-Sartor

- Cualquiera de ellos estará capacitado para responder las preguntas que Usted tenga.

Yo (nombre y apellidos) ____ declaro que:
- He leído la hoja de información del estudio que se me ha entregado
- He podido hacer preguntas sobre el estudio
- He recibido suficiente información sobre el estudio
- He estado informado por (nombre del investigador) __________________________

Comprendo que mi participación es voluntaria y que puedo retirarme del estudio:
- Cuando quiera
- Sin dar explicaciones
- Sin que ello repercuta en la atención médica recibida

Declaro haber sido informado que mis datos serán tratados de conformidad con lo que establece la L.O. de 13 de diciembre y de Protección de Datos de Carácter Personal (artículo 3, punto 6 del RD 223/2004).

Consiento que los datos clínicos referentes a mi enfermedad sean almacenados en un fichero automatizado que podrá ser manejado exclusivamente con fines científicos.

____

Por tanto: doy mi consentimiento a participar en el estudio que se me propone

Firma del médico responsable | Firma del voluntario

Dr/a.: | Sr/a.:
Núm. Col.:
Fecha:

____

He decidido no autorizar a participar en el estudio que se me ha propuesto

Firma del médico responsable | Firma del voluntario

Dr/a.: | Sr/a.:
Núm. Col.:
Fecha:

Sant Josep de la Muntanya,12 I 08024 Barcelona I Telèfon 933 674 100 I Fax 933 674 266
www.parcdesalutmar.cat
