# Supplementary material for: The Retornus-2 study: impact of respiratory muscle training in subacute stroke patients with dysphagia, study protocol of a double-blind randomized controlled trial
Source: Trials. 2021 Jun 25;22:416. doi: 10.1186/s13063-021-05353-y (PMC8229262; doi:10.1186/s13063-021-05353-y)
Supplement: Supplementary file 2 — Additional file 2. Ethical Approval Document [file 13063_2021_5353_MOESM2_ESM.pdf]

CONFIRMATION

I hereby certify that "Parc de Salut MAR - Clinical Research Ethics Committee" has reviewed and approved

- ✓ The clinical study entitled: *"Estudio RETORNUS-2: Impacto del entrenamiento respiratorio en la función deglutoria en pacientes con disfagia secundaria a ictus"*.

In which Dr. ANNA GUILLÉN SOLÀ is the principal investigator.

- ✓ And the corresponding patient information sheet and informed consent form.

Under the reference number: 2016/6796/I

At the meeting of: June 21st , 2016

Mª Teresa Navarra Alcrudo
Secretary
Ethics Committee – Parc de Salut MAR

Barcelona, June 22nd, 2016

CEIC – Parc de Salut MAR
Dr. Aiguader, 88 I 08003 Barcelona I Telèfon 93 316 06 77 I Fax 93 316 06 36
ceic-psmar@imim.es | www.parcdesalutmar.cat
