# Supplementary material for: The Retornus-2 study: impact of respiratory muscle training in subacute stroke patients with dysphagia, study protocol of a double-blind randomized controlled trial
Source: Trials. 2021 Jun 25;22:416. doi: 10.1186/s13063-021-05353-y (PMC8229262; doi:10.1186/s13063-021-05353-y)
Supplement: Supplementary file 4 — Additional file 4. Funding Documentation. [file 13063_2021_5353_MOESM4_ESM.pdf]

Dear Editor,

Find attached two original reports regarding the resolution of the call for grants of the Strategic Health Action 2013-2016, from the Institute of Health Carlos III (ISCIII) and co-funded by the European Regional Development Fund (ERDF).

The first of the reports corresponds to the project and the research team evaluation, being obtaining the favorable final qualification required to obtain funding.

The second report shows the total funding awarded (50215 €), divided by concepts and annuities.

General information of the study:

PROJECT: **Estudio RETORNUS-2: Impacto del entrenamiento respiratorio en la función deglutoria en pacientes con disfagia secundaria a ictus**

MAIN INVESTIGATOR: Anna Guillen-Sola

EXPEDIENT: **PI16/01081**

APPLICANT CENTER: Fundació Institut Mar d'Investigacions Mèdiques

CENTER DIRECTOR: Institut Hospital del Mar d'Investigacions Mèdiques (IMIM)

Fundació IMIM

Servei de Recerca

IMIM ((Hospital del Mar Medical Research Institute)

Biomedical Research Park of Barcelona

Dr. Aiguader, 88 / 08003 Barcelona

Tel: 00 34 93 316 05 76
